# MiSeq Sequencing of Salmonella enterica subsp. *houtenae* Isolates from a Dog Treated for Hind-Limb Paresis

**DOI:** 10.1128/MRA.00655-20

**Published:** 2020-08-06

**Authors:** Mary L. Krath, Andrew E. Hillhouse, Sara V. Little, Sara D. Lawhon

**Affiliations:** a Department of Veterinary Pathobiology, College of Veterinary Medicine & Biomedical Sciences, Texas A&M University, College Station, Texas, USA; b Texas A&M Institute for Genome Sciences and Society, College of Veterinary Medicine & Biomedical Sciences, Texas A&M University, College Station, Texas, USA; Loyola University Chicago

## Abstract

The genomes of three clinical isolates of Salmonella enterica subsp. *houtenae* were sequenced using an Illumina MiSeq instrument. These isolates came from the urine and cerebrospinal fluid of a dog treated for hind-limb paresis with immunosuppressive drugs. S. enterica subsp. *houtenae* has also been implicated in brain infections in humans.

## ANNOUNCEMENT

Salmonella enterica subsp. *houtenae* was originally isolated from a cockatiel in 1978 and has been recovered from a variety of animals, including mammals, birds, reptiles, and amphibians (1–9). Although infrequently isolated from humans, S. enterica subsp. *houtenae* has been reported in association with meningitis and brain abscesses, primarily in children and immunocompromised adults (10–14). The isolates sequenced here were originally isolated from urine (isolates 36GH and 76AB, collected in August and October 2019, respectively) and cerebrospinal fluid (isolate 38CD, collected in August 2019) from a 5-year-old spayed female boxer dog that was being treated with immunosuppressive drugs for hind-limb paresis.

Patient specimens were spread onto a blood agar plate (BAP) with Trypticase soy agar and 5% sheep’s blood and incubated at 35°C ± 2°C in an atmosphere supplemented with 5% CO_2_. The organisms were identified using matrix-assisted laser desorption–time of flight (MALDI-TOF) mass spectrometry using the Biotyper (Bruker Daltonics, Billerica, MA) with flexControl v3.4 build 135.14 software. The National Veterinary Services Laboratory analyzed the organisms for serotyping and determined them to be S. enterica subsp. *houtenae* serotype 43:z4,z32:− (15).

Isolates were stored at −80°C in *Brucella* broth supplemented with 10% glycerol and revived for sequencing by inoculating an aliquot of the frozen bacteria onto a BAP. They were grown in 5 ml lysogeny broth overnight at 37°C with aeration. DNA isolation from 1-ml aliquots of the overnight cultures was performed according to the manufacturer’s (Macherey-Nagel) protocol using Macherey-Nagel type B bead tubes and lysis buffer from the NucleoMag tissue DNA kit with a Qiagen TissueLyser machine. DNA quality was verified using a genomic DNA TapeStation run (Agilent).

Default parameters were used for the following steps, and manufacturer’s instructions were followed, unless otherwise stated. Illumina libraries were prepared using the Illumina Nextera DNA Flex library preparation kit and sequenced on an Illumina MiSeq v2 2 × 250-bp kit. All data were uploaded to BaseSpace v5.39, NativeApp.Core v0.9.0.2 (Illumina) for run monitoring, FASTQ file generation, demultiplexing, and adapter trimming. The sequencing output of paired-end read sets comprised 251 bp for all three isolates. Isolate 36GH had 1,616,328 reads, isolate 38CD had 1,411,316 reads, and isolate 76AB had 1,652,672 reads. This resulted in approximately 92× coverage, 80× coverage, and 94× coverage, respectively.

Assembly was conducted using the -careful parameter on SPAdes v3.13.0 (16). The assemblies of isolates 36GH, 38CD, and 76AB contained 77, 74, and 90 contigs, respectively. The genome lengths were 4,580,467, 4,579,974, and 4,586,535 bp, respectively, and the *N*_50_ contig lengths were 220,987, 265,007, and 265,007, respectively, with an approximate GC content of 51% for all three isolates. The Benchmarking Universal Single-Copy Orthologs (BUSCO) score was used to analyze completeness, with a score of 100% for all three isolates based on the *Enterobacteriales* OrthoDB v9 data set (17, 18).

The three isolates from this patient were all identified to be Salmonella enterica subspecies *houtenae* serovar 43:z4,z32:− using SeqSero, in agreement with the National Veterinary Laboratory report (15, 19). The three isolates were submitted for NCBI average nucleotide identity (ANI) analysis, which confirmed their identification (20). Ribosomal multilocus sequence typing found that the 54 *Salmonella* ribosomal genes (*Salmonella* spp. carry two copies of *rpmJ*) were identical in these three isolates, supporting the idea that all three isolates were clonal (21). This supports the hypothesis that all three isolates from this patient, two from the urinary bladder collected at different times and one from the cerebrospinal fluid, were the same organism and that this was a case of disseminated salmonellosis.

### Data availability.

The whole-genome shotgun sequences and raw reads for 36GH, 38CD, and 76AB can be found under the BioProject accession number PRJNA600881. The genome sequences for 36GH (TAMU36GH), 38CD (TAMU38CD), and 76AB (TAMU76AB) are available under the GenBank accession numbers JAAAGG000000000, JAAAGF000000000, and JAAAGE000000000, respectively. The raw reads are available under the SRA accession numbers SRR10876206, SRR10876207, and SRR10876208, respectively.
